# 

**Published:** 1882-02

**Authors:** 


CONTENTS.

| | |
|---|---|
| Mad Dogs | 55 |
| Taking Cold | 57 |
| Chronic Diarrhea | 58 |
| Diet of the Japanese | 59 |
| Plants that Eat Animals | 60 |
| The Horse on the Prairie | 62 |
| Gossip | 63 |
| Life in the London Slums | 64 |
| The Siamese White Elephants | 65 |
| The Needle of the Compass | 67 |
| The Dying | 69 |
| Hygiene | 70 |
| How People Take Cold | 77 |
| Running Up-Stairs | 79 |
| Tobacco: its Use and End | 80 |
| A Sad Reflection | 83 |
| Consumption | 85 |
| Reading | 97 |
| Hot-Air Furnaces | 98 |
| Longevity | 100 |
| Military Ants of the Amazon | 101 |
| Their "Jibbenenosey" | 103 |
| An Orange Wrapping in Florida | 105 |

